# References values and predictive factors for thyroid volume in pregnant women

**DOI:** 10.20945/2359-3997000000656

**Published:** 2023-06-19

**Authors:** Lisette Leal Curí, María de las Mercedes Rubén Quesada, Daysi Antonia Navarro Despaigne, Esther Jequín Savariego, Lisandro Chávez González, Drissa Bina Konaré, Erick Robles Torres

**Affiliations:** 1 Universidad de Ciencias Médicas de La Habana Cuba Universidad de Ciencias Médicas de La Habana, Cuba; 2 Instituto Nacional de Endocrinología Departamento de Docencia e Investigación La Habana Cuba Departamento de Docencia e Investigación, Instituto Nacional de Endocrinología, La Habana, Cuba; 3 Instituto Superior de Ciencias Básicas y Preclínicas “Victoria de Girón” Departamento de Ciencias de la Computación La Habana Cuba Departamento de Ciencias de la Computación, Instituto Superior de Ciencias Básicas y Preclínicas “Victoria de Girón”, La Habana, Cuba; 4 Hospital Universitario “Manuel Fajardo” Departamento de Imagenología La Habana Cuba Departamento de Imagenología, Hospital Universitario “Manuel Fajardo”, La Habana, Cuba

**Keywords:** Thyroid volume, pregnancy, reference values

## Abstract

**Objective:**

Thyroid volume varies within each population according to different clinical and biochemical factors and can change during pregnancy. The present investigation was aimed to determine the reference values for thyroid volume in pregnant women and their predictive factors.

**Materials and methods:**

A cross-sectional study was carried out with 360 healthy pregnant women. The following variables were examined: maternal age, gestational age, skin color, current smoking status, parity, use of iodinated supplements, body mass index, thyrotropin, total and free thyroid hormones, thyroglobulin, antithyroid antibodies, chorionic gonadotropin, cholesterol and triglycerides.

**Results:**

The mean thyroid volume was 5.3 ± 1.3 mL, 5.4 ± 1.6 mL and 5.6 ± 2.5 mL in the first trimester, second trimester and third trimester, respectively. The reference interval was 2.47-9.49 mL in the first trimester, 3.17-9.01 mL in the second trimester, and 3.00-12.38 mL in the third trimester. Free triiodothyronine and triglycerides were predictors of thyroid volume (corrected R^2^ = 0.12; p = 0.000).

**Conclusion:**

This study is the first to determine the reference values for thyroid volume and its predictive factors in pregnant women from Cuba, a Caribbean island with sustainable elimination of iodine deficiency disorders.

## INTRODUCTION

In the diagnosis of thyroid diseases, determination of thyroid volume is very useful. Screening for thyroid dysfunction in pregnancy is currently recommended in women with risk factors, such as goiter and (according to some authors) small thyroid (1,2). The World Health Organization (WHO) considers thyroid ultrasound the method of choice for goiter detection (3).

During pregnancy, a woman's thyroid function undergoes important changes due to several physiological mechanisms. First, serum concentrations of thyroxine binding globulin (TBG) double or triple due to the effect of estrogens (4). In addition, at the beginning of pregnancy, human chorionic gonadotropin (hCG) stimulates the gland, as it is structurally homologous to thyrotropin (TSH) (5). Moreover, the intense activity of placental deiodinase leads to an increase in the catabolism of thyroxine (T4) and triiodothyronine (T3) (1,4).

Changes in thyroid physiology are also reflected in the growth of the gland to meet the hormonal demands of the mother and the fetus. According to some reports, during pregnancy, the thyroid significantly increases in volume, while other researchers observed slight or no growth; these differences are largely due to the genetic and environmental factors of each population, especially iodine intake (6-9). Reports of gland volume in pregnant women from different regions describe means between 8 and 12 mL (7,8,10,11).

Investigations of the thyroid volume of pregnant women are scarce, and their conclusions are heterogeneous (7,8,12). In Cuba, a National Program was established in 2002 to ensure the mandatory iodization of salt for human consumption. As a result, in 2005, the country was declared to have sustainable elimination of iodine deficiency disorders, and systematic monitoring of the program was maintained, with sustained salt iodization with average levels of 18-25 ppm (13,14). Data on the iodine status of pregnant women in Cuba are limited, although an adequate intake in this subpopulation is reported (15). However, there are no reference intervals for thyroid volume in pregnant Cuban women. Therefore, the present study aimed to determine the reference values for thyroid volume in participating pregnant women and their predictive factors.

## MATERIALS AND METHODS

A cross-sectional study was carried out in women with a clinical and ultrasound diagnosis of pregnancy who lived in Plaza municipality, Havana, in the period between March 2015 and February 2020.

Thyroid volume was determined in a sample of these women who were recruited for another research project to establish the reference values for thyrotropin and thyroid hormones in healthy pregnant women, with a minimum number of 120 women studied per trimester (16).

Plaza municipality was selected because it is the area where the National Institute of Endocrinology is located. Three of the seven polyclinics in this municipality were chosen by simple random sampling: *Moncada*, *Vedado* and *19 de Abril*. Pregnant women from the selected health areas were recruited, stratified by trimester of pregnancy, according to the size of the population in each health center. As a result, 108 women from the *Moncada* polyclinic, 159 from the *Vedado* polyclinic and 93 from the *19 de Abril* polyclinic were included.

### Exclusion criteria

We excluded women with multiple pregnancy, those with a personal or family history of thyroid disease, those with conditions that affect the metabolism of iodine or thyroid hormones (kidney failure, liver failure, intestinal malabsorption) and those taking drugs that interfere with thyroid physiology (glucocorticoids, amiodarone, phenytoin, carbamazepine, lithium, furosemide). Subsequently, women diagnosed with nodules on thyroid ultrasound and those whose anti-thyroid antibodies were positive were excluded.

### Procedures

The enrolled pregnant women underwent an interview to obtain data such as maternal age, gestational age, skin color, current smoking status, parity, body mass index (BMI) at the beginning of pregnancy (the latter taken from the clinical history of prenatal care) and consumption of nutritional supplements containing iodine. In Cuba, the prescription of nutritional supplements with iodine to pregnant women is not standardized. Pregnant women who consumed these supplements were self-medicated, with preparations containing between 150 and 220 μg of iodine.

A blood sample was also taken to determine levels of TSH, total and free T4, total and free T3, thyroglobulin (Tg), hCG, cholesterol and triglycerides as well as the presence of antibodies against thyroid peroxidase (TPOAb) and against thyroglobulin (TgAb). Thyroid ultrasound was performed to measure the volume of the gland and identify the presence of nodules by the same experienced specialist. All these procedures were carried out in the morning hours.

### Biochemical and hormonal determinations

Measurements of TSH, total and free T4, total and free T3, thyroglobulin, TgAb and TPOAb were performed using commercially available kits from IZOTOP (Institute of Isotopes Ltd, Hungary). TSH was measured by immunoradiometric assay with a functional sensitivity of 0.07 mIU/mL and inter- and intra-assay coefficients of variation (CVs) of 2.0% and 1.19%, respectively. Total and free T4 and T3 were determined by direct radioimmunoassay; for total T4, the functional sensitivity was 7 nmol/L, and the inter- and intra-assay CVs were 2.8% and 2.3%, respectively; for total T3, the functional sensitivity was 0.22 nmol/L, and the interassay and intra-assay CVs were 4.1% and 2.98%, respectively; for free T4, the functional sensitivity was 0.7 pmol/L, and the inter- and intra-assay CVs were 2.83% and 0.94%, respectively; and for free T3, the functional sensitivity was 0.58 pmol/L, and the interassay and intra-assay CVs were 4.76% and 2.2%, respectively. Tg was measured by immunoradiometric assay with a functional sensitivity of 0.022 ng/mL and inter- and intra-assay CVs of 1.7% and 1.8%, respectively. hCG was determined by electrochemiluminescence immunoassay for quantitative in vitro determination of the sum of hCG plus b-hCG in serum and plasma (Cobas, Roche Diagnostics International Ltd.) with a functional sensitivity of 0.6 mIU/L.

Manufacturer reference ranges were 0.30-4.00 mIU/L for TSH, 55-170 nmol/L for total T4, 10-22 pmol/L for free T4, 1.25-3.03 nmol/L for total T3, 1.9-5.7 pmol/L for free T3, and 2-70 ng/mL for Tg. TgAb and TPOAb were measured by radioimmunoassay and were considered positive when these levels were greater than 100 mIU/mL (for TgAb) and greater than 25 mIU/mL (for TPOAb).

Total cholesterol and triglycerides in serum were determined by the enzymatic method. Functional sensitivity was 0.07 mmol/L for total cholesterol, with a CV ≤ 5% and a reference interval of 2.9-5.2 mmol/L. In the case of triglycerides, the functional sensitivity was 0.09 mmol/L, and the CV was ≤ 5%, with a reference interval of 0.46-1.60 mmol/L.

Thyroid volume was measured with an Aloka SSD 1400 device (Mitaka-Shi, Tokyo, Japan) with a 7.5-MHz linear transducer and was calibrated before starting the study. The pregnant woman lay supine, with her neck extended and a pillow behind her shoulders. The Brunn equation (17) recommended by the WHO was used to calculate thyroid volume in the population (3) as follows:

Thyroid volume = anteroposterior diameter (cm) × medial-lateral diameter (cm) × cranial-caudal diameter (cm) × 0.479.

To calculate the total volume, the measurements of both lobes were added. The presence or absence of nodules or cysts was also identified. All measurements were carried out by the same specialist with experience in carrying out this procedure.

### Statistical analysis

SPSS version 21.0 for Windows was used for statistical analysis. To determine the normality of distributions of the variables studied, the Kolmogorov-Smirnov test was performed, and homoscedasticity was verified using the Levene test. All variables had a normal distribution except for total T4 levels in the first trimester and TSH, thyroglobulin, and hCG levels in all trimesters.

Quantitative variables are expressed as the means and standard deviations, and qualitative variables are expressed as frequencies and percentages. One-way analysis of variance was used to compare the quantitative variables among pregnancy trimesters, with the exception of TSH, thyroglobulin and hCG, with which the Kruskal-Wallis test was used as they did not have a normal distribution. To explore differences in qualitative variables by trimester, the chi-square test was used. Thyroid volume percentiles were determined by weeks of gestation, and to establish the reference interval by trimester, the 2.5th percentile of the original variable was taken as the lower limit and the 97.5th percentile was taken as the upper limit. The construction of percentile curves for thyroid volume was carried out using the R package GAMLSS in R version 14.0.

The associations of thyroid volume with clinical and biochemical variables were explored using Pearson's correlation analysis for quantitative variables (maternal and gestational age, square root of TSH, total and free thyroid hormones, cholesterol, triglycerides, and the logarithm of Tg and hCG). Volume means were compared among skin color categories using one-way analysis of variance and according to smoking status, parity, and the use of iodinated supplements using Student's t tests. Based on the correlations among variables and the analysis of variance results, multiple linear regression analyses were carried out to evaluate the joint and independent ability of factors to explain the variability in the dependent variable. In each case, the corresponding contrasts were made to verify the required assumptions of normality and homoscedasticity of variance. To assess collinearity, the condition index was used. An index value above 15 indicated possible collinearity, and a value above 30 indicated high collinearity. For all analyses, a statistical significance threshold of α = 0.05 was applied.

### Ethics

The included pregnant women participated voluntarily in this study. They were provided with information on the objectives of the study and the procedures involved, and their written informed consent was obtained. The results of this research were used only for scientific and care purposes, and the confidentiality of the data was maintained. This project was approved by the Research Ethics Committee of the National Institute of Endocrinology of Cuba (Code I 070LH 1506).

## RESULTS

The general characteristics of the pregnant women studied are summarized in Table 1. Maternal age and pregestational body mass index were similar among the three trimesters. In each group, pregnant women who were white skin color, nulliparous, nonsmokers and did not consume iodinated supplements accounted for the highest proportion; there were no significant differences among groups. Thyroglobulin was lower in pregnant women in the second trimester, and hCG was higher in women in the first trimester. Thyroid volume, cholesterol and triglycerides were higher in women in later trimesters.

**Table 1:** Characteristics of the pregnant women studied

Variable		First trimester (n = 120)	Second trimester (n = 120)	Third trimester (n = 120)	Test statistic	p
Age (years)		28.4 ± 5.0	27.9 ± 4.8	28.0 ± 5.1	F = 0.328	0.72^a^
Gestational age (weeks)		11.4 ± 1.3	18.3 ± 3.3	30.9 ± 3.9	-	-
Pregestational BMI (kg/m^2^)		24.0 ± 4.9	24.6 ± 5.0	24.0 ± 3.8	F = 0.465	0.63^a^
Skin color						
	White	75 (61.5)	71 (59.2)	76 (63.3)	χ^2^ = 1.806	0.77^b^
	Black	17 (14.0)	14 (11.6)	16 (13.3)		
	Mixed	30 (24.6)	35 (29.2)	28 (23.4)		
Smoking status						
	Smoker	10 (8.2)	3 (2.5)	12 (10.0)	χ^2^ = 6.638	0.16^b^
	Nonsmoker	112 (91.8)	117 (97.5)	108 (90.0)		
Parity						
	Nulliparous	75 (61.5)	76 (63.3)	75 (62.5)	χ^2^ = 0.09	0.96^b^
	Multiparous	47 (38.5)	44 (36.7)	45 (37.5)		
Iodinated supplement use						
	Yes	17 (13.9)	18 15.0)	12 (10.0)	χ^2^ = 6.488	0.59^b^
	No	105 (86.1)	102 (85.0)	108 (90.0)
Thyroglobulin (ng/mL)		12.1 (9.3)	8.4 (9.3)	11.1 (11.6)	χ^2^ = 15.06	0.001^c^
hCG (mIU/mL)		55 880 (4 989)	26 580 (2 512)	15 120 (1 569)	χ^2^ = 84.64	0.000^c^
Thyroid volume (mL)		5.3 ± 1.3	5.4 ± 1.6	5.6 ± 2.5	F = 1.932	0.05^a^
Cholesterol (mmol/L)		4.3 ± 0.7	5.3 ± 1.0	6.0 ± 1.3	F = 65.402	0.000^a^
Triglycerides (mmol/L)		1.2 ± 0.5	1.6 ± 0.6	2.1 ± 0.8	F = 56.434	0.000^a^

Qualitative variables are represented as frequency (%), and quantitative variables are represented as the mean (± standard deviation) or median (interquartile range), as appropriate.

a One-way analysis of variance

b χ^2^ test

c Kruskal-Wallis test

**Figure 1:**
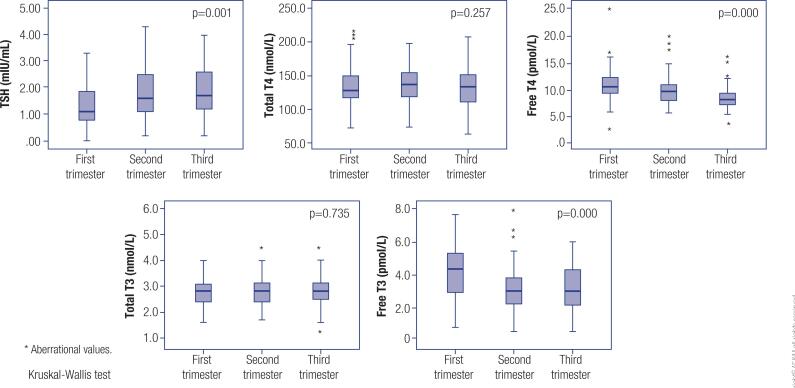
Variables related to thyroid function by trimester of pregnancy.

The distribution of the variables related to thyroid function according to trimester is shown in Figure 1. Mean TSH levels were lowest in women in the first trimester of pregnancy (1.2 mIU/L) and higher in those in the second (1.7 mIU/L) and third trimester (1.8 mIU/L; p = 0.001).

The mean total T4 level was not significantly different between pregnant women in the first trimester (125.0 nmol/L) and those in the second (135.4 nmol/L) and third trimesters (131.5 nmol/L). Free T4 levels were lower in women in later trimesters (10.9 pmol/L in the first trimester, 9.8 pmol/L in the second trimester and 8.5 pmol/L in the third trimester; p = 0.000).

Regarding T3, the levels of its total fraction showed a mean of 2.7 nmol/L in pregnant women in the first trimester and 2.8 nmol/L in those in the second and third trimester. Free T3 levels were higher in women in the first trimester (4.2 pmol/L) than in those in the second and third trimester, with means of 3.0 pmol/L and 3.1 pmol/L, respectively (p = 0.000).

Table 2 shows the percentile distribution of thyroid volume according to trimester of pregnancy. The reference interval in the first trimester was between 2.47 and 9.49 mL; in the second trimester, the range was between 3.17 and 9.01 mL; and in the third trimester, the range was between 3.00 and 12.38 mL.

**Table 2:** Percentiles of thyroid volume according to trimester of pregnancy

Trimester	Percentile				
	2,5	25	50*	75	97,5
First	2.47	4.45	5.28	6.58	9.49
Second	3.17	4.41	5.36	6.58	9.01
Third	3.00	4.77	5.55	6.00	12.38

* One-way ANOVA (p = 0.107)

From the average values of thyroid volume in each trimester, it appears that there was not a progressive effect of time on volume; indeed, statistical analysis corroborated this conclusion. A post hoc analysis revealed a statistical power of 0.2; that is, the possibility of detecting a real effect of time on thyroid volume was only 20%.

**Figure 2:**
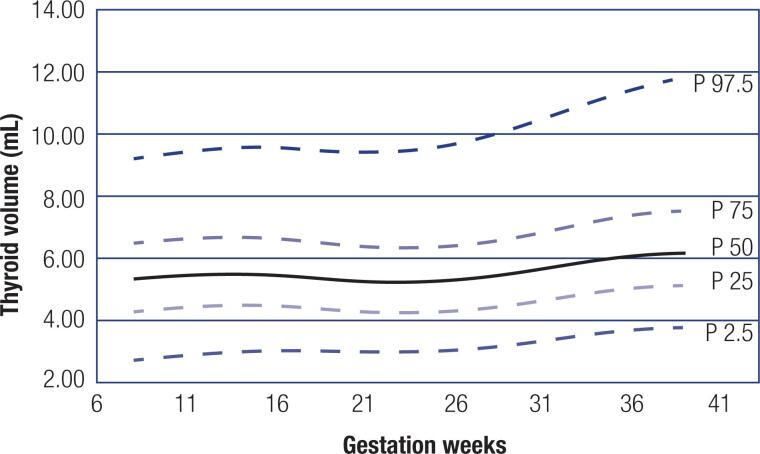
Percentile curves for thyroid volume according to weeks of gestation.

Figure 2 shows the thyroid volume percentile curves according to weeks of gestation. After 24 weeks of gestation, the curve increased until the end of pregnancy, when the greatest thyroid volume was reached in these pregnant women.

The association of each individual clinical and biochemical factor with thyroid volume was explored in pregnant women, and the following variables were significantly correlated: free T3 (r = 0.19; p = 0.00), Tg (r = 0.11; p = 0.05), triglycerides (r = 0.26; p = 0.00) and hCG (r = −0.20; p = 0.01). The remaining variables (maternal age, gestational age, skin color, current smoking status, parity, use of iodinated supplements, BMI, TSH, total T4 and T3, free T4 and cholesterol) were not significantly associated with thyroid volume.

The significant variables from the univariate analysis were included in a multivariate regression analysis with thyroid volume as the dependent variable. The results indicated that two models were able to explain the variance in thyroid volume of pregnant women. The best fitting model (adjusted R^2^ = 0.12; p = 0.000; condition index < 15) indicated that only free T3 and triglycerides were good predictors of thyroid volume.

## DISCUSSION

Some studies have supported a goitrogenic effect of pregnancy due to a deficient intake of iodine (6,18,19). In addition, during pregnancy, there is a significant expansion of plasma and extracellular volume, especially in the third trimester, which leads to increased blood flow in different maternal organs, including the thyroid (20).

However, in the present study, the thyroid volume of pregnant women was lower than that described in another Cuban study of nonpregnant women (6.4 mL) (21). This may be due to the lower mean age of the pregnant women in the present study, as a positive correlation between age and gland size has been reported (22).

The trend toward increased thyroid size after 24 gestational weeks in the present study is consistent with the findings of other studies (7,8). Around the third trimester, there is a peak in serum estrogen concentrations, with an increase in the vasculature of the uterus and placenta. Elevated serum levels of these hormones increase hepatic synthesis and decrease TBG catabolism due to the higher content of sialic acid in the carrier protein, thereby increasing total thyroid hormone concentrations in the blood, particularly total T4, since its affinity for this hormone is greater than that of T3 (1,4,23). This also increases iodine requirements; if iodine intake is insufficient, an increase in thyroid size could occur. However, in the present study, a progressive increase in thyroid volume from the first to the third trimester was not observed. This could be due to the cross-sectional nature of the study, as women in the different trimester subgroups may have had different iodine intake. It is possible that a larger sample size may have revealed a clearer effect.

Studies have shown an association between thyroid volume and thyroid function (11,12). The pregnant women in the present study showed only a correlation of thyroid volume with free T3, which could indicate thyroid autoregulation prior to potential insufficient intake of iodine in at least some pregnant women. The lack of an association between thyroid volume and the rest of the variables may be because these women had no personal or family history of thyroid disease and thus had normal thyroid function.

Studies on the relationship between the volume of the thyroid and metabolic syndrome, in which alterations occur in the metabolism of carbohydrates and lipids, have reported that the common denominator is insulin resistance (24-26). In the present study, triglycerides were positively associated with thyroid volume, consistent with findings of other authors (24,25).

This is the first study to determine the reference values for thyroid volume and its predictive factors in pregnant women from Cuba, a Caribbean island with sustainable elimination of iodine deficiency disorders. However, urinary iodine concentrations were not assessed, which is a limitation due to the important role of iodine deficiency in goitrogenesis. Additionally, thyroid volume may vary depending on the genetic, nutritional, and environmental characteristics of each population; thus, these reference values may not be generalizable to all regions of the country. However, these new data may facilitate more precise diagnosis of thyroid disease in pregnant women in the study region.
